# Declines in Perceived Control and Subsequent Passive Suicidal Ideation Among Older Adults

**DOI:** 10.1093/geroni/igaf122.2702

**Published:** 2025-12-31

**Authors:** Kallisse Dent, Leah Richmond-Rakerd

**Affiliations:** University of Michigan, Ann Arbor, Michigan, United States; University of Michigan, Ann Arbor, Michigan, United States

## Abstract

Perceived control tends to decline in older adulthood, while suicide rates tend to increase. More information is needed about how declines in perceived control may shape suicide risk over time. In the present study, we tested whether declines in perceived control were longitudinally associated with passive suicidal ideation (PSI). As suicide rates tend to be highest among older white men, we also tested effect modification by demographic factors. Data were from the Health and Retirement Study, a nationally-representative longitudinal cohort of adults aged 51+. Decline in perceived control was measured across a 4-year period (2010-2014 or 2012-2016) using 10 items that indexed perceived constraints on personal control and perceived mastery. Passive suicidal ideation was assessed using the Composite International Diagnostic Interview-Short Form in the subsequent wave (2016 or 2018). Logistic regression was used to test associations of decline in perceived control with PSI, with interaction terms used to test effect modification by age, sex, race, ethnicity, and education. Analyses accounted for complex survey design and adjusted for demographics, prior psychiatric diagnosis, and PSI at baseline. Preliminary data analyses found that 43.6% of older adults experienced a decline in perceived control across a 4-year period. Older adults who experienced a decline in perceived control were at higher risk for PSI (OR = 1.79, 95% CI: 1.37-2.32). There was no significant effect modification by demographic factors. Declines in perceived control precipitate passive suicidal ideation. Perceived control may be an important target for suicide prevention among older adults.

